# Magnetic properties of Fe_3_O_4_ antidot arrays synthesized by AFIR: atomic layer deposition, focused ion beam and thermal reduction

**DOI:** 10.3762/bjnano.9.164

**Published:** 2018-06-11

**Authors:** Juan L Palma, Alejandro Pereira, Raquel Álvaro, José Miguel García-Martín, Juan Escrig

**Affiliations:** 1 Departamento de Ciencias Básicas, Centro de Ingeniería y Desarrollo Sustentable, Facultad de Ingeniería, Universidad Central de Chile, Santa Isabel 1186, 8330601 Santiago, Chile; 2 Center for the Development of Nanoscience and Nanotechnology (CEDENNA), 9170124 Santiago, Chile; 3 Departamento de Física, Universidad de Santiago de Chile (USACH), Avda. Ecuador 3493, 9170124 Santiago, Chile; 4 Instituto de Micro y Nanotecnología, IMN-CNM, CSIC (CEI UAM+CSIC), Isaac Newton 8, 28760 Tres Cantos, Madrid, Spain

**Keywords:** antidot arrays, atomic layer deposition, focused ion beam, magnetic properties, thermal reduction

## Abstract

Magnetic films of magnetite (Fe_3_O_4_) with controlled defects, so-called antidot arrays, were synthesized by a new technique called AFIR. AFIR consists of the deposition of a thin film by atomic layer deposition, the generation of square and hexagonal arrays of holes using focused ion beam milling, and the subsequent thermal reduction of the antidot arrays. Magnetic characterizations were carried out by magneto-optic Kerr effect measurements, showing the enhancement of the coercivity for the antidot arrays. AFIR opens a new route to manufacture ordered antidot arrays of magnetic oxides with variable lattice parameters.

## Introduction

Magnetic antidots, magnetic thin films with periodic arrays of holes, are currently an important topic for both the fundamental understanding of low-dimensional magnetism and a broad range of applications, such as a new generation of electronic devices [1], sensors [2], ultra-high density recording media – due to the absence of the superparamagnetic limit as there are no isolated magnetic islands – [3], and magnonics and spintronic devices [4–5]. The presence of the ordered non-magnetic holes induces a demagnetization field distribution that changes the magnetization switching mechanisms [6], acting as pinning centers for domain walls [7], enhancing coercivity compared to that of the continuous film [8–12], and affecting the magnetic properties of the film [13–19]. Thus, the antidot geometry can also be used to tailor the coercivity and the frequencies of the ferromagnetic resonance modes [20–22].

It is well known that there are numerous techniques for attaining magnetic antidot arrays such as e-beam [6,16], UV [23] and colloidal [24] lithography, porous anodic alumina [25–26], block copolymer templates [27], nanochannel glass [28] and focused ion beam (FIB) patterning [29–30]. Recently, we have proposed the fabrication of disordered antidot arrays through the thermal reduction of thin films synthesized by atomic layer deposition (ALD) [31–33]. Due to the self-limited growth of material, ALD allows to control the thickness of the films with high precision [34]. The holes arise because of a dewetting process of the sample [35], which depends on its geometric and magnetic parameters as well as on the conditions of synthesis and thermal reduction. Hence, the holes are quite inhomogeneous and appear in disordered form on the sample. Thus, in this article we are interested in introducing a new procedure for obtaining antidot arrays with new properties. The technique is called AFIR (from ALD + FIB + reduction), and it consists of the deposition of a thin film by ALD, the generation of holes by means of FIB, and the thermal reduction of the antidot arrays. AFIR opens a new route to manufacture ordered antidot arrays of oxides with variable lattice parameters, arrays that have not been synthesized by other techniques. As a proof of concept, we will investigate the magnetic properties of Fe_3_O_4_ antidot arrays that have never been fabricated until now. As magnetic antidots have been successfully used to preferentially capture magnetic nanoparticles within the holes [36], and as Fe_3_O_4_ is a biocompatible material, such new Fe_3_O_4_ antidot arrays are of interest for the future development of nano-scale biosensors.

## Experimental

**Figure 1:**
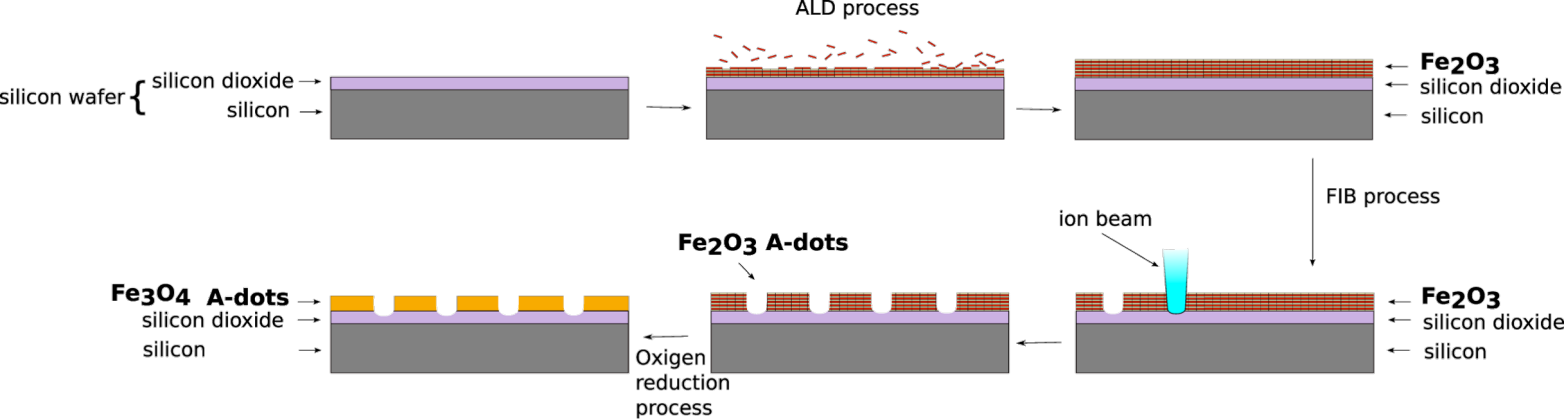
Outline of the AFIR process. Si(100) wafers with a native layer of SiO_2_ were coated with Fe_2_O_3_. Antidot arrays were directly etched in the continuous films of Fe_2_O_3_ using an IonLine FIB machine. The Fe_2_O_3_ antidot arrays are thermally reduced whereby Fe_3_O_4_ antidot arrays are obtained.

Figure 1 shows the outline of the AFIR process. Si(100) wafers with a native layer of SiO_2_ were coated with hematite (Fe_2_O_3_) in a Savannah S100 ALD reactor from Ultratech operated at 200 °C in stop/exposure-mode. The ferrocene (FeCp_2_) was held in a stainless-steel container (bottle) heated to 90 °C to ensure sufficient vapor pressure. The pulse times of ferrocene and ozone in the FeCp_2_/O_3_ cycle were 2 s and 0.2 s, respectively; the exposure and pump times were 5 s and 15 s, respectively. As one of the reactants is ozone with a volume concentration of about 10%, we have used an OzoneLab generator Ol80W/FM100V. During the process, a flow of 20 sccm of nitrogen has been maintained. As a proof of concept we have deposited 2250 ALD cycles to obtain a Fe_2_O_3_ film of 27 nm thickness. We have obtained a deposition rate lower than that obtained in [31], but it is important to note that the substrate used was different in both cases.

Antidot arrays were directly etched in the continuous film using an IonLine FIB machine with 30 keV Ga ions, and opening of 30 μm, 17.5 pA ion current and a dose of 30 mC/cm^2^. The dwell time was chosen to be sure that the ion beam completely perforated the Fe_2_O_3_ film and that the hole diameter was quite homogeneous, so at least 20 nm of the substrate were also etched. These antidot arrays are then placed into a furnace GSL-1100X from MTI Corporation, which has a controlled atmosphere of hydrogen (4%) balanced with argon (96%) at an overpressure of 400 mbar with a set temperature of 430 °C, for 4 h [32–33]. This process allows for the conversion of Fe_2_O_3_ to Fe_3_O_4_, which exhibits a strong magnetic signal.

Atomic force microscopy (AFM) measurements have been performed using a Bruker Dimension Icon microscope operating in non-contact mode and commercial AFM probes (Nanosensors, type PPP-FM), while scanning electron microscopy (SEM) images have been obtained using a Zeiss EVO MA10 microscope. The thickness of the thin films was determined using an alpha-SE ellipsometer from J. A. Wollam, while X-ray diffraction (XRD) measurements were performed using a Bruker D8 system with Cu Kα radiation (λ = 0.15406 nm), in a 2θ range between 10° and 90° at a sweep rate of 0.02°·s^−1^. Longitudinal MOKE hysteresis loops of the antidot arrays were obtained using a NanoMOKE3 from Quantum Design with the applied magnetic field applied parallel to the substrate plane and reaching a maximum value of 1.5 kOe. The laser spot was placed into each antidot array and, in order to check that the spot was located in the right position, the longitudinal reflectivity was measured. Magnetic field was applied along the 0° and 45° directions when measuring the square arrays (i.e., the first and second neighbors directions, respectively) and along 0° and 30° direction when measuring the hexagonal arrays (first and second neighbors directions in this case, respectively). Additionally, one of the samples was measured every 15°.

## Results and Discussion

**Figure 2:**
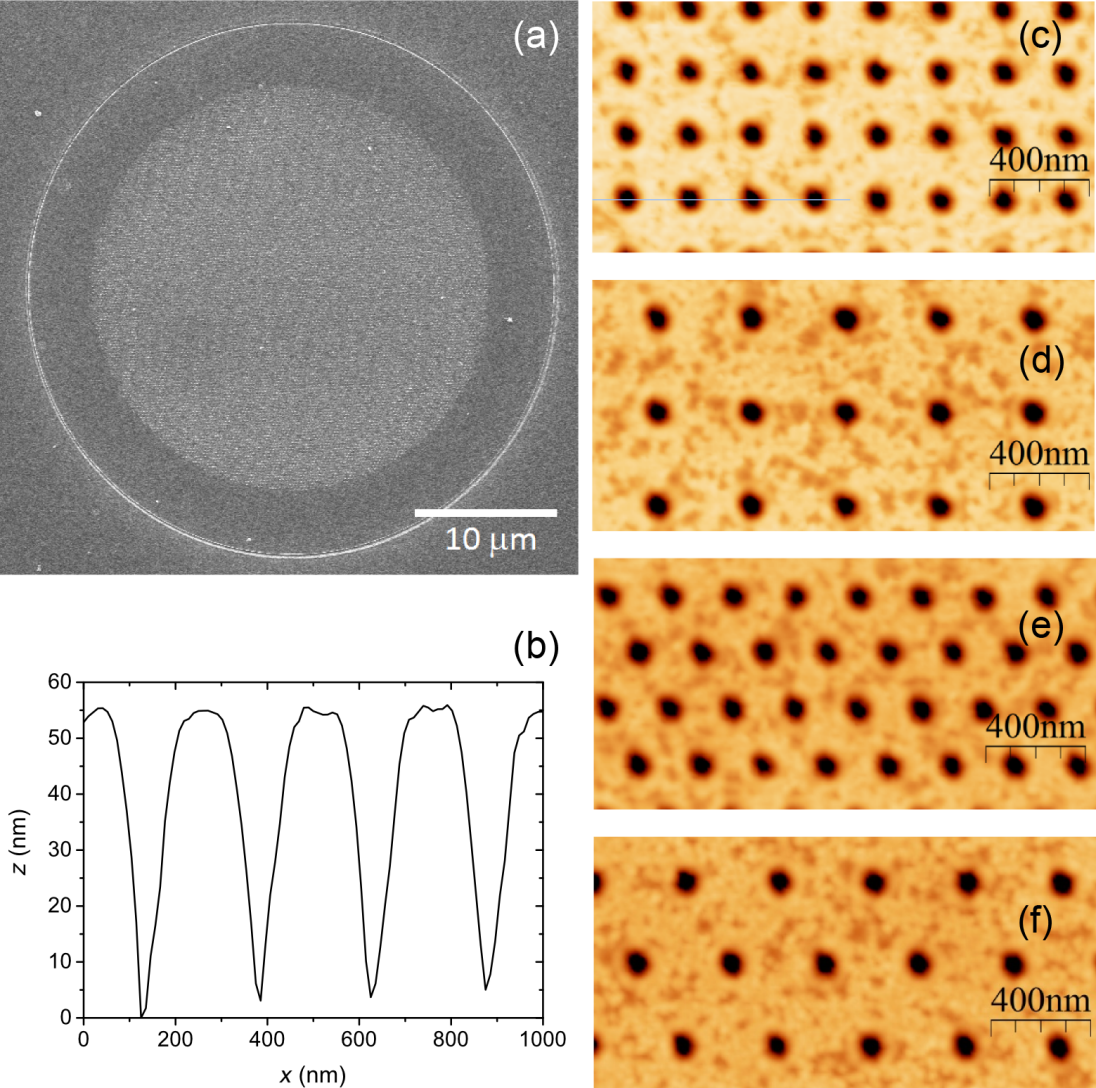
(a) SEM image of the antidot array patterned on the film with 27 nm thickness. (b) Profile obtained from the AFM image corresponding to a square array with a hole spacing of 240 nm, in particular along the blue line of image c. Square array with a lattice constant of 240 (c) and 360 nm (d). Hexagonal arrays with a lattice constant of 240 (e) and 360 nm (f).

Figure 2a shows the SEM image of a representative Fe_2_O_3_ antidot array patterned on the film with 27 nm thickness. After patterning the antidot array has the shape of a circle of 30 μm diameter, which is surrounded by a circular trench of 40 μm diameter that allows one to isolate the magnetic signal from the rest of the magnetic film. The trench was etched using a 20 mC/cm^2^ ion beam dose. Moreover, for the sake of comparison, regions confined by a trench but without any pattering, i.e., Fe_2_O_3_ disks with 40 μm diameter, were also prepared.

Four different antidot arrays have been patterned, combining two symmetries (hexagonal and square order) with two different lattice constants (240 and 360 nm), as shown in Figure 2c–f. The geometric parameters of the antidots are obtained from profiles obtained from AFM images like the one shown in Figure 2b, corresponding to a square array with a hole spacing of 240 nm. It is verified that the depth of the etched holes is at least 50 nm, i.e., not only the 27 nm thick Fe_2_O_3_ film is etched but also at least 20 nm of the substrate, in agreement with previous works to be sure of the uniformity of the magnetic antidots [30]. From this profile, the diameter of the antidots is determined as the full width at half maximum of the hole, and it is measured to be of the order of 70 nm. Moreover, it seems that the holes have a conical structure, but this is due to the convolution with the AFM tip, which has a pyramidal geometry with 25° slope.

Once the thermal reduction is performed, the morphology is preserved and the Fe_3_O_4_ antidots are obtained. Only a slight reduction in roughness is produced: In the AFM measurements performed in the thin film regions, the roughness of the initial Fe_2_O_3_ film was 0.8 nm, whereas that of the final Fe_3_O_4_ film was 0.6 nm.

**Figure 3:**
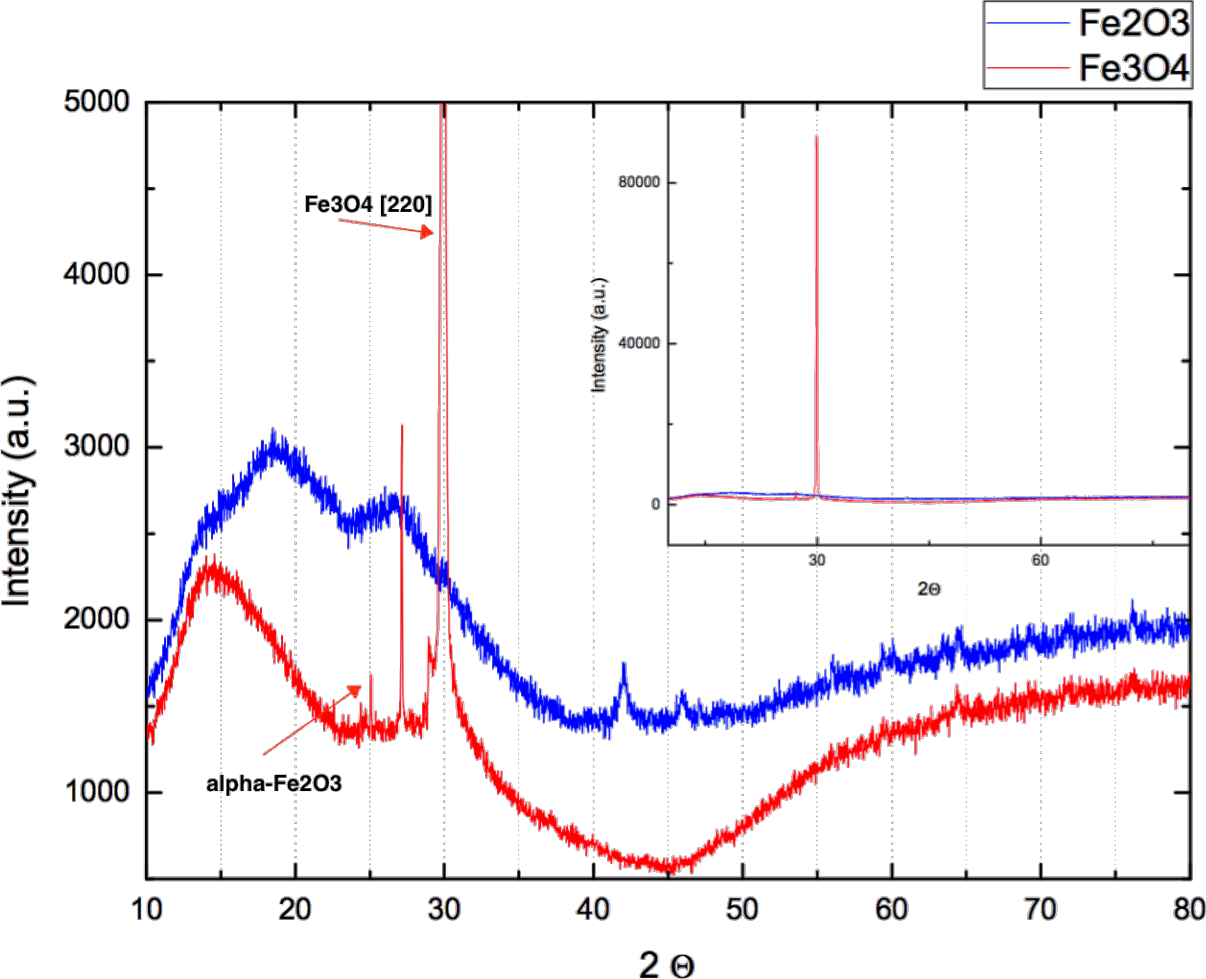
XRD patterns of the as-deposited Fe_2_O_3_ film (blue curve) and the Fe_3_O_4_ film (red curve) after the thermal reduction process.

Figure 3 displays the XRD patterns of as-deposited Fe_2_O_3_ film (upper curve) and the Fe_3_O_4_ film (lower curve) after thermal reduction, for 2250 ALD cycles. The Fe_2_O_3_ film pattern exhibits one peak at approximately 43°. The Fe_3_O_4_ film pattern exhibits two peaks corresponding to the planes (112) and (200), which according to ICSD card No. 01-075-1609 corresponds to an orthorhombic structure. It is important to note that a small trace of Fe_2_O_3_ still exists in the sample, indicating that the thermal reduction process was not able to convert all Fe_2_O_3_ to Fe_3_O_4_. However, the magnetic signal measured later will confirm the transformation from a paramagnetic to a ferrimagnetic sample.

Let us discuss the morphology of the Fe_3_O_4_ antidots. In order to efficiently modify the magnetic properties of a magnetic film by digging holes in it, the diameter of the holes has to be of the same order of magnitude as the domain wall width. Assuming Bloch-type domain walls, the width *W* is given by 
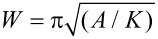
 where *A* is the exchange constant and *K* is the magnetic anisotropy. Taking common values of *A* = 15.3 × 10^−12^ J/m [37] and *K* = 2.1 × 10^4^ J/m^3^ [38], *W* = 84 nm is obtained, so a hole diameter of 70 nm is a good choice. Concerning the lattice parameter, the close proximity of neighboring holes may induce some issues since regions affected by the tail of the Gaussian-like section of the ion beam may overlap [29–30]. In order to avoid such effects, we have chosen lattice parameter values at least three times larger than the hole diameter.

**Figure 4:**
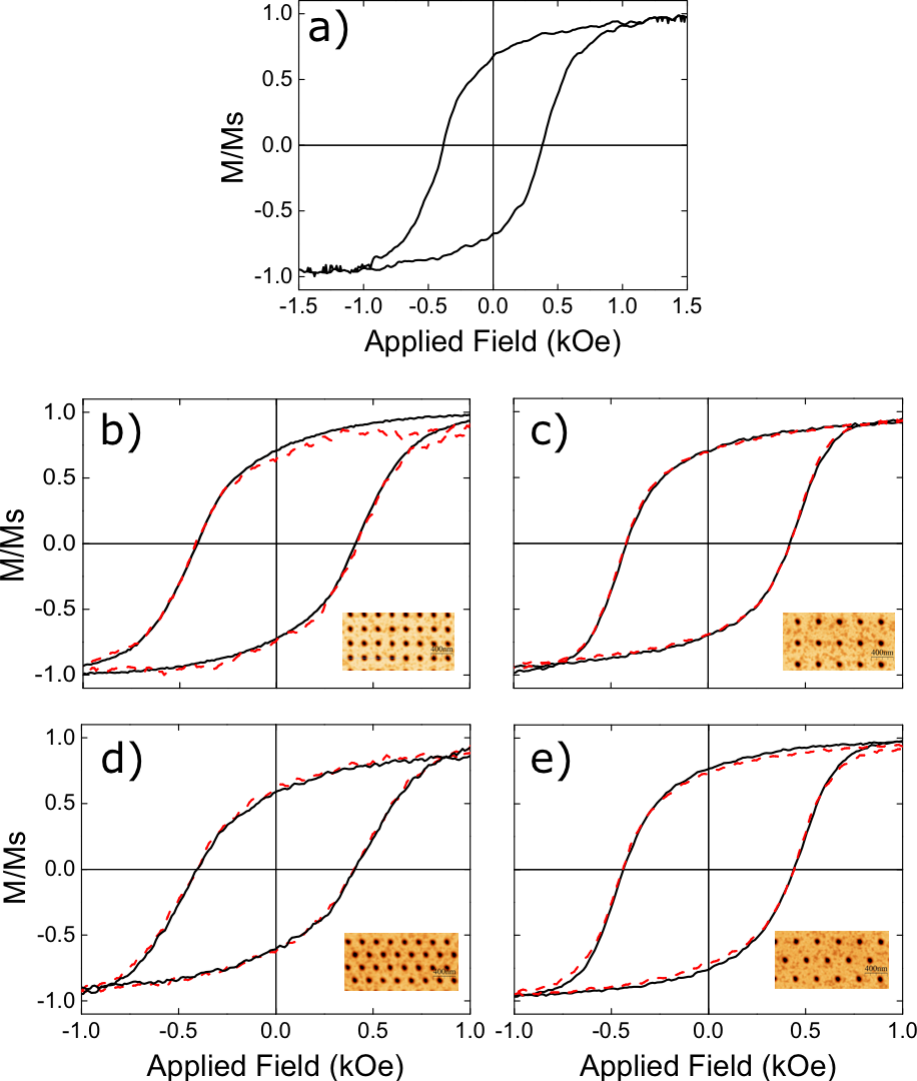
Central region of the hysteresis curves for the antidot arrays obtained from a 27 nm thick film. (a) Pristine thin film; (b, c) square arrays with lattice parameters of 240 and 360 nm, respectively; (d, e) hexagonal arrays with lattice parameters of 240 and 360 nm, respectively. Solid black lines correspond to a field applied in the direction of first neighbors, while the red dashed lines to a field applied in the direction of the second neighbors.

**Figure 5:**
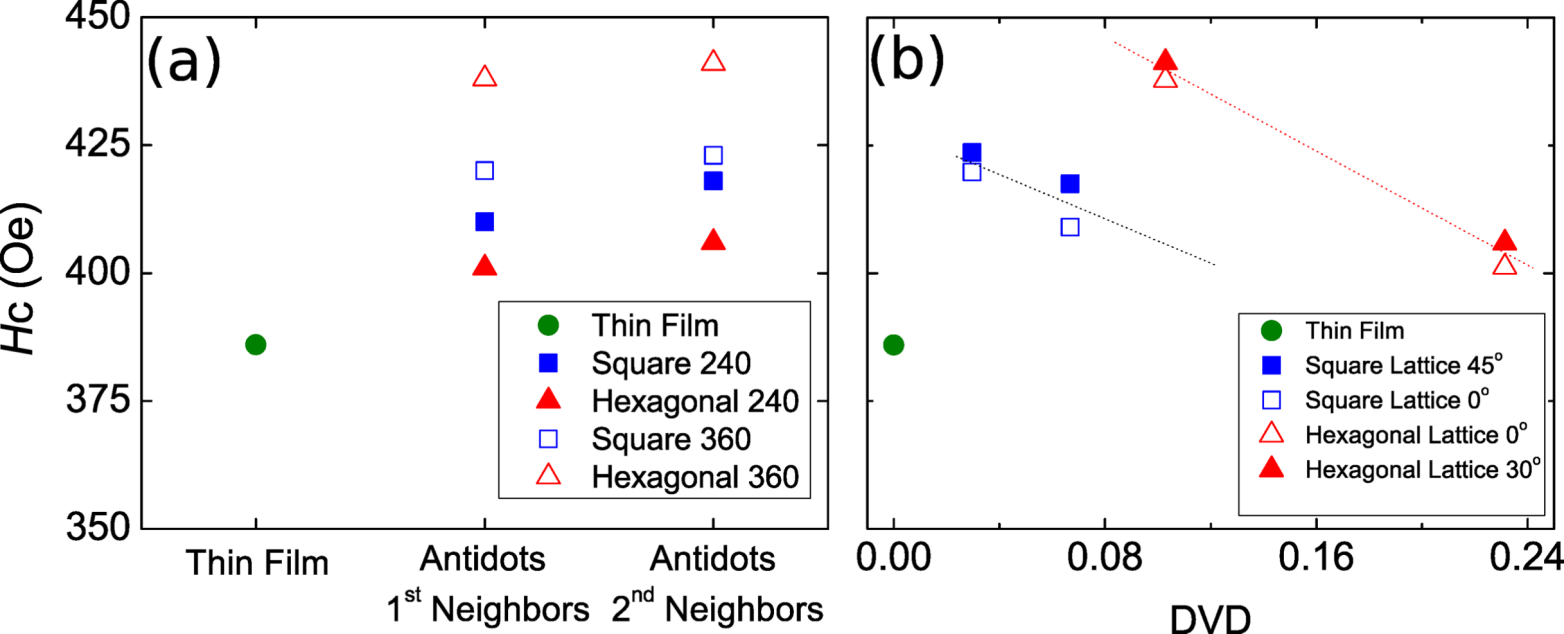
(a) Coercivity of the initial thin film and of the square (blue squares) and the hexagonal (red triangles) arrays, considering two different lattice parameters, and applying the external magnetic field along the first and second neighbors directions. (b) Coercivity as a function of the defect volume density (DVD).

According to the magnetic measurements, the initial Fe_2_O_3_ film is paramagnetic at room temperature, whilst after thermal transformation the obtained Fe_3_O_4_ film is ferrimagnetic. Figure 4 shows the representative hysteresis curves for a Fe_3_O_4_ sample with 27 nm thickness, for the thin film as well as for the antidot arrays. Figure 4a shows the loop of the thin film: It is worth noting that this thin film synthesized by AFIR exhibits a coercivity of about 380 Oe, which is higher than that exhibited by thin films synthesized by other techniques [31–33]. This fact can be ascribed to internal defects induced during the last step of the AFIR technique, i.e., the thermal reduction process needed to obtain the ferrimagnetic Fe_3_O_4_ film. In spite of this fact, the antidot arrays exhibit enhanced coercivity, as shown in Figure 4b,c for those with square order and in Figure 4d,e for those with hexagonal order. This enhancement can be attributed to the additional pinning of the magnetic domain walls produced by the holes [7,12,30]. At first sight it is observed that both coercivity and remanence of the arrays with larger lattice parameters are slightly larger than those of the arrays with smaller lattice parameters (i.e., the curves exhibit a wider and more vertical shape), regardless of whether we treat square or hexagonal arrays. Small differences exist between the loops obtained with the external magnetic field applied along the first and second neighbors directions, as detailed for the coercivity in Figure 5. The coercivity is enhanced for all the antidot arrays, in agreement with the results obtained with antidots fabricated with other routes [8–12].

From Figure 5a it is observed that the coercivity increases with increasing the lattice parameter of the array and by applying the magnetic field along the direction of the second-neighbor holes. This means that the coercivity increases with increasing space between the holes in the direction in which the external magnetic field is applied. Of course, this does not continue indefinitely, since the limiting case in which the holes are infinitely separated can be considered as a thin film, the coercivity of which is lower than that of an antidot pattern. More studies would be needed to obtain the threshold value of the lattice parameter at which the tendency changes. It is important to point out that the coercivities obtained by the AFIR technique are almost twice as high as those obtained with other techniques, considering similar geometrical and magnetic parameters [39].

From a magnetic viewpoint each nanohole may be considered as a defect since they act as pinning centers for the domain wall motion during magnetization reversal. If *a* is the lattice constant (nearest neighbor center-to-center distance) and *d* is the hole diameter, the defect volume density (DVD), which is the ratio of the surface covered by holes to the total surface, is given by DVD_sq_ = (π/4)·(*d*/*a*)^2^ for the square arrays and by 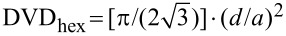 for the hexagonal arrays. In order to highlight the influence of DVD on the pinning strength, Figure 5b shows all obtained coercivity values as a function of the DVD. For both symmetries, there is a monotonic decrease of coercivity as the DVD increases, a behavior opposite to that observed in arrays of antidots obtained from other synthesis techniques [30], which may be associated with the thermal reduction process of AFIR.

**Figure 6:**
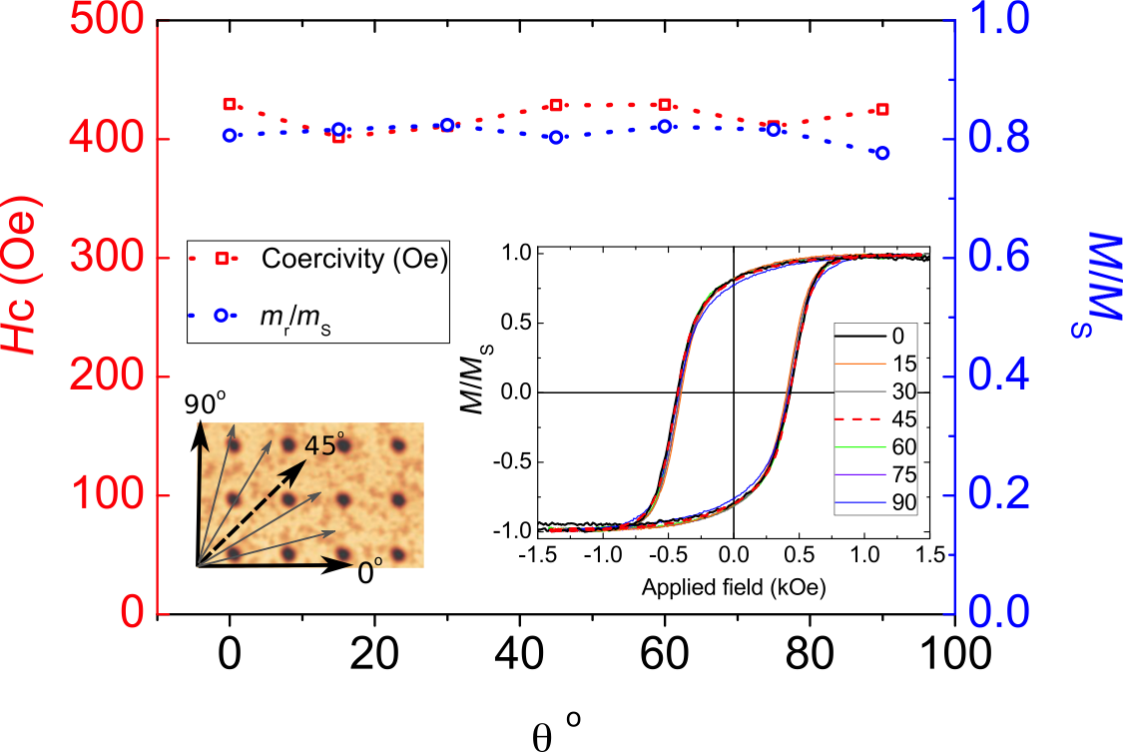
Coercivity (red squares) and normalized remanence (blue dots) as a function of the angle θ at which the external magnetic field is applied for a square array with lattice parameter 360 nm.

Figure 6 shows the coercivity and normalized remanence as a function of the angle at which the external magnetic field is applied for a square array with lattice parameter 360 nm. From this measurement it is clearly observed that the coercivity increases if the external magnetic field is applied for angles in which pores are found and decreases for angles at which there are no pores. This is mainly because the pores act as pinning zones during the magnetization reversal process. The remanence exhibits the opposite behavior with values close to 0.8.

## Conclusion

In conclusion, we have demonstrated the technical feasibility of manufacturing magnetic antidot arrays using a new technique called AFIR. As a proof of concept we have synthesized thin films of Fe_2_O_3_ that were then imprinted with a pattern of antidots by FIB, and finally thermally reduced to obtain the first Fe_3_O_4_ antidot arrays. We have observed that the coercivity of the antidot arrays is enhanced compared to that of the synthesized films. This new technique paves the way to manufacture square and hexagonal antidot arrays of magnetic oxides among other geometries, and with variable lattice parameters.
